# Soft Zr-doped TiO_2_ Nanofibrous Membranes with Enhanced Photocatalytic Activity for Water Purification

**DOI:** 10.1038/s41598-017-01969-w

**Published:** 2017-05-09

**Authors:** Jun Song, Xueqin Wang, Jianhua Yan, Jianyong Yu, Gang Sun, Bin Ding

**Affiliations:** 1 State Key Laboratory for Modification of Chemical Fibers and Polymer Materials, College of Materials Science and Engineering, Donghua University, Shanghai, 201620 China; 2 Key Laboratory of Textile Science & Technology, Ministry of Education, College of Textiles, Donghua University, Shanghai, 201620 China; 3 Nanofibers Research Center, Modern Textile Institute, Donghua University, Shanghai, 200051 China

## Abstract

Self-standing photocatalytic membranes constructed from TiO_2_ nanofibers hold great promise in environmental remediation; however, challenges still remained for the poor mechanical properties of polycrystalline TiO_2_ nanofibers. Herein, soft Zr-doped TiO_2_ (TZ) nanofibrous membranes with robust mechanical properties and enhanced photocatalytic activity were fabricated *via* electrospinning technique. The Zr^4+^ incorporation could effectively inhibit the grain growth and reduce the surface defects and breaking point of TiO_2_ nanofiber. The as-prepared TZ membranes were composed of well-interconnected nanofibers with a high aspect ratios, small grain size and pore size, which exhibited good tensile strength (1.32 MPa) and showed no obvious damage after 200 cycles of bending to a radius of 2 mm. A plausible bending deformation mechanism of the soft TZ membranes was proposed from microscopic single nanofiber to macroscopical membranes. Moreover, the resultant TZ membranes displayed better photocatalytic performance for methylene blue degradation compared to a commercial catalyst (P25), including high degradation degree of 95.4% within 30 min, good reusability in 5 cycles, and easiness of recycling. The successful preparation of such fascinating materials may open up new avenues for the design and development of soft TiO_2_-based membranes for various application.

## Introduction

The water pollution induced by the excessive sewage discharge from industrial manufacturing has threatened the ecological and human health in modern society. Various treatment methods such as precipitation, adsorption, filtration, chemical oxidation, and biological degradation, *etc*, have been developed to remove organic contaminations from wastewater^1–3^. As an environmentally friendly green technique, photocatalysis has been considered as one of the most promising strategies for environmental remediation. TiO_2_ is a most common semiconductor photocatalyst applied in degradation of organic pollutants due to its low cost, nontoxicity, and long-term stability against photocorrosion^4, 5^. In the past few decades, a number of strategies have been focused on how to improve the activity by modifying the properties of TiO_2_. Recently, it has been reported that the introduction of small amount of Zr could improve the thermal stability of anatase phase and TiO_2_ surface properties, thus enhancing the photocatalytic activity^6, 7^. Although Zr-doped TiO_2_ photocatalysts have been prepared by several wet chemical methods including sol-gel, hydrothermal, co-precipitation and so on refs 8 and 9, the majority of the obtained photocatalysts are generally in nanoparticles form, which are difficult to recycle due to the suspended dispersive properties of nanoparticles in water. Immobilizing nanoparticles photocatalysts on supporting materials, such as polymer, ceramic, and carbon, is an effective way to avoid the aggregation of nanoparticles, and simplify the separation and reclaiming procedure^10–13^. Nevertheless, previous studies have demonstrated that the immobilization of TiO_2_ nanoparticles on substrates may lead to a large part of the particles inaccessible in use, which seriously decreased the photcatalytic efficiency^10, 14^. Therefore, developing a facile and effective approach to create TiO_2_-based materials with high photocatalytic reactivity and good recyclability should be of great interest for practical applications.

Recently, electrospinning has become a simple and versatile method for preparing nanofibrous materials with many attractive properties, involving large specific surface area (SSA), high porosity, and interspatial connectivity^15–17^. Owing to these advantageous features, electrospun TiO_2_ nanofibers possess high reactivity, and their fibrous structure makes it possible to conveniently separate and reclaim such materials from water after environmental remediation. However, their polycrystalline nature usually causes mechanically fragile, such a situation hinders their further developments^2, 18, 19^. Up to now, only a few soft electrospun TiO_2_-based fibrous membranes have been prepared, such as SiO_2_-TiO_2_ and CeO_2_-TiO_2_ fibrous membranes^20, 21^. In our previous study, we reported a soft Y^3+^-doped TiO_2_ fibrous membranes with a tensile strength of 472.5 kPa^19^; however, this membranes showed reduced photocatalytic activity towards MB compared to pure TiO_2_ nanofibers, due to an increase in electron-hole recombination after doping with Y^3+^ ions. Thus, it is still an imperative and challenging issue to develop soft TiO_2_ photocatalytic membranes with simultaneously enhanced mechanical properties and photocatalytic performance.

In this work, we demonstrated a novel soft TZ fibrous membranes with robust mechanical properties and efficient photocatalytic activity fabricated via electrospinning technique. The tensile strength, softness and photocatalytic performance of TZ membranes were thoroughly investigated basing on fiber morphology, crystal structure, and porous structure, which were modulated by regulating the Zr doping content. A probable soft mechanism of the polycrystalline TZ fibrous membranes was also proposed. Significantly, the resultant TZ membranes showed excellent photocatalytic performance in the degradation of MB under UV light irradiation, and the soft photocatalytic membranes could be easily extracted from solution after utilization, which could serve as a promising photocatalysts for environmental remediation in the future.

## Results

### Morphology and structure analysis

**Figure 1:**
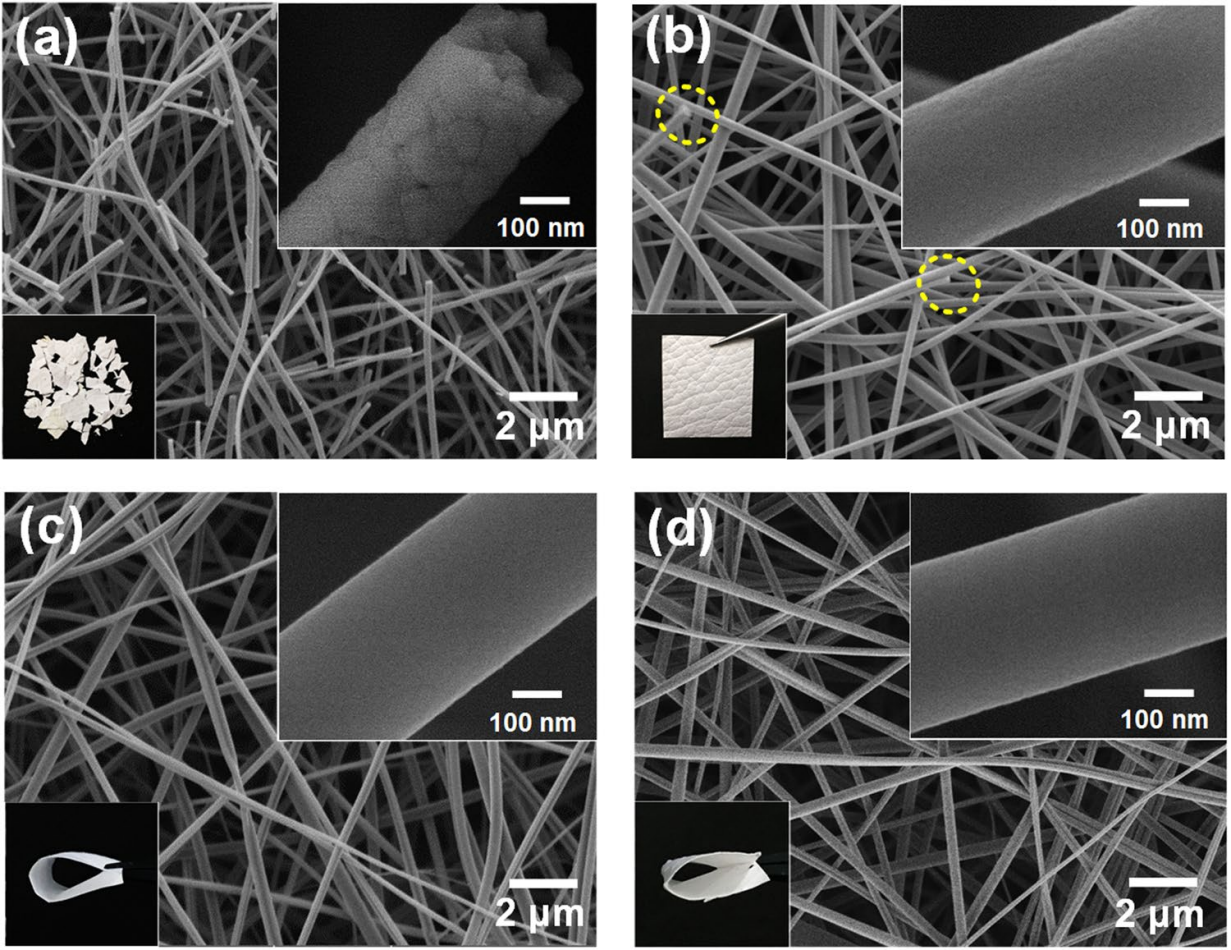
FE**-**SEM images of (**a**) TiO_2_, (**b**) TZ-5, (**c**) TZ-10, and (**d**) TZ-20 fibrous membranes. The insets are the optical images and magnified images of the corresponding fibrous membranes.

The mechanical properties and photocatalytic performance of materials are generally closely associated with the morphologies and structure of nanofibrous membranes, thus, the morphologies, crystalline structure and porous structure were firstly analysed. Figure 1a presents the typical image of pure TiO_2_ membrane, which clearly shows that the membrane consisted of a large number of broken nanofibers, and many surface defects can be obviously observed in the magnified FE-SEM image of single fiber. Meanwhile, this membrane was in a fragments form and showed extreme fragility. Interestingly, when the TiO_2_ fibers were doped with 5 mol% Zr, the defects and breakages on the fibers decreased sharply, and the membrane exhibited good self-standing behaviour (Fig. 1b). As the doping Zr content increased to 10 and 20 mol%, the fibers became smooth and continuous (Fig. 1c and d) with an ultrahigh aspect ratio (>1000) (Figure S3). The resultant TZ-10 and TZ-20 membranes could be bent 200 times without any cracks (Fig. 1c and d). In addition, it can be found that the average diameters of TiO_2_ fibers increased regularly from 304 to 371 nm with the increment of Zr contents, this could be ascribed to the enhanced viscosity and decreased conductivity of precursor solutions (Table S1). These results suggested that the perfect morphology of TiO_2_ nanofibers could be obtained by Zr doping, which have positive effect on the softness of TiO_2_ membranes.

**Figure 2:**
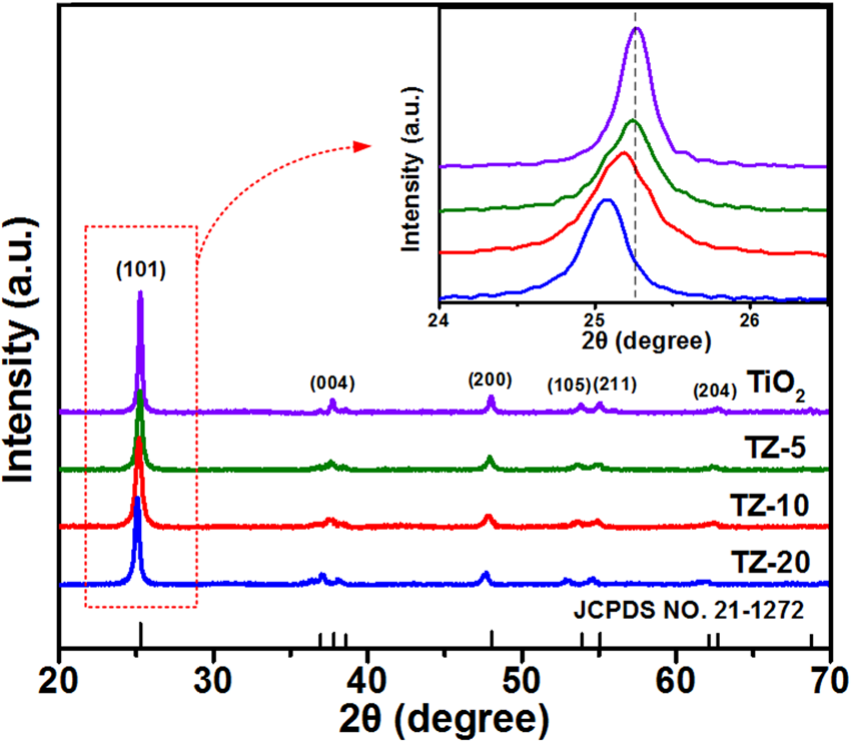
XRD patterns of TiO_2_, TZ-5, TZ-10, and TZ-20 nanofibrous membranes. The inset shows the enlargement of the XRD peaks for the (101) plane.

The crystalline phases of pristine TiO_2_ and TZ membranes were investigated by XRD as presented in Fig. 2. All the diffraction peaks could be assigned to the anatase phase of TiO_2_ (JCPDS no. 21–1272)^19^, and no other peak corresponding to Zr compound could be identified. This implies that Zr was distributed homogeneously within the TiO_2_ nanofibers, and the concentration of Zr species was below the detection limit of XRD^22^. Furthermore, it is clear that the (101) anatase peak for TZ membranes gradually shifted to lower angles with the increase of Zr^4+^ ion concentration (inset in Fig. 2), suggesting that the doping Zr^4+^ ions entered into the TiO_2_ lattice by replacing the lattice Ti^4+^ ions^23, 24^, which could also be verified by the Zr 3d XPS spectrum of TZ-10 membranes (Figure S4). Since the ionic radius of Zr^4+^ ions (0.72 Å) is slightly larger than that of Ti^4+^ ions (0.65 Å)^25^, the lattice parameters and cell volume of TiO_2_ increased gradually after Zr^4+^ ions doping (Table S2). In addition, the average crystallite sizes of pure TiO_2_ and TZ nanofibers were calculated using the Scherrer equation and the results are listed in Table S2. In contrast with TiO_2_ nanofibers (29.1 nm), the TZ-5, TZ-10, and TZ-20 nanofibers possessed a relative smaller crystalline size of 21.8, 17.5, and 22.4 nm, respectively, due to the incorporation of Zr can provide dissimilar boundaries and inhibit the grain growth of TiO_2_^26, 27^. Moreover, the TZ-10 nanofibers still exhibited an anatase phase with small crystalline size of 26.6 nm even when the annealing temperature was 800 °C (Figure S5 and Table S3), suggesting the Zr doping significantly enhanced the thermal stability of TiO_2_ nanofibers. However, excessive Zr doping would decrease the amount of phase boundaries and result in the coarsening of TiO_2_ nanocrystals^20^. Hence, choosing the suitable doping concentration of Zr is very important for controlling the grain size of TiO_2_ nanofibers.

**Figure 3:**
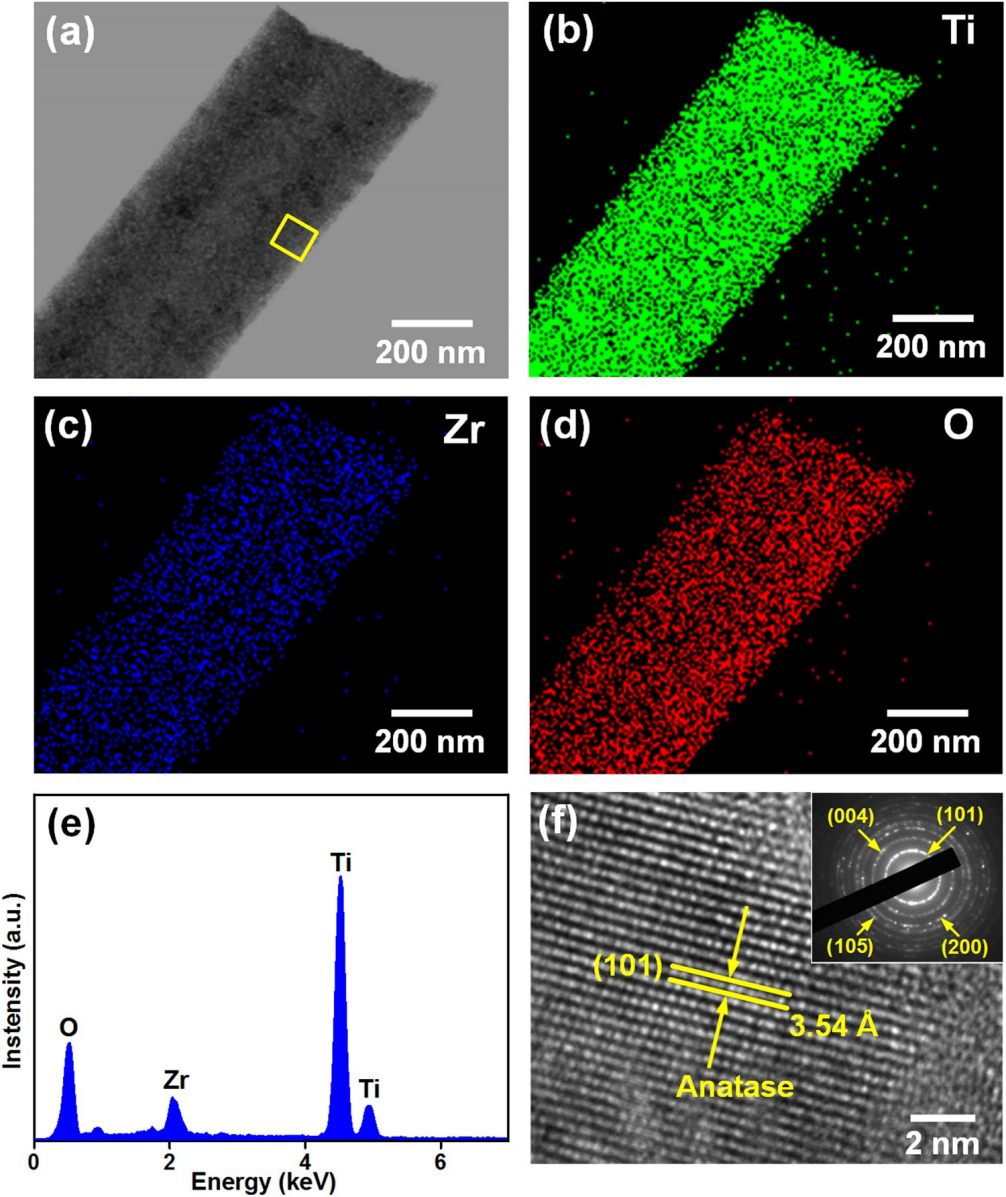
(**a**) TEM image of TZ-10 nanofiber. (**b**–**d**) Elemental maps of Ti, Zr and O by analyzing the fiber in (**a**). (**e**) EDS spectrum of TZ-10 nanofiber. (**f**) HR-TEM image of the marked area in (**a**). The inset in (**f**) is the corresponding SAED pattern of TZ-10 nanofiber.

To further investigate the soft TZ nanofiber internal structure, we performed STEM and EDS on the TZ-10 nanofibers. The STEM images presented in Fig. 3a–d verified the homogeneous distribution of Ti, Zr, and O elements in the nanofiber at the nanoscale. Additionally, the corresponding EDS spectrum (Fig. 3e) further confirmed the co-existence of Ti, Zr, and O elements, and the XPS spectrum analysis (Figure S6) revealed that the content of Zr, Ti, and O were 3.0%, 29.6%, and 67.4%, respectively, which was very close to the theoretical value of TZ-10 nanofibers. The HR-TEM image of the selected area in Fig. 3a revealed that the lattice fringe spacing of (101) crystallographic plane for TZ-10 (3.54 Å) (Fig. 3f) was slightly larger than that for pristine anatase TiO_2_ (3.52 Å)^23, 28^, implying that Zr^4+^ ions was doped into anatase TiO_2_ lattice in substitutional mode^23^, which was in good agreement with previous XRD analysis. The SAED pattern illustrated that the TZ-10 fiber remained the polycrystalline anatase nature without any additional diffraction of second phases.

**Figure 4:**
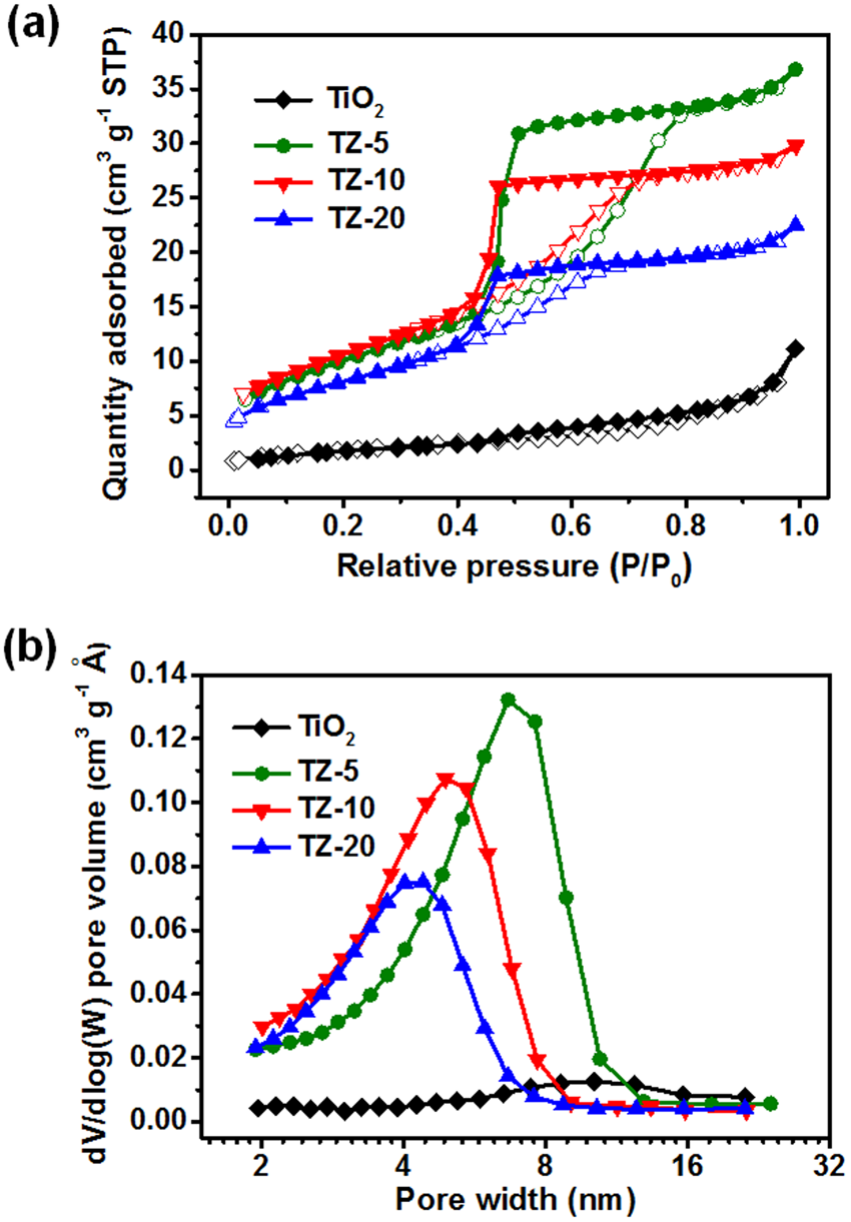
(**a**) N_2_ adsorption-desorption isotherms of TiO_2_, TZ-5, TZ-10, and TZ-20 nanofibrous membranes. (**b**) Pore distribution analysis of the relevant membranes using the BJH method.

The porous textures of as-prepared materials were analysed by N_2_ adsorption-desorption measurement. As depicted in Fig. 4a, all the nanofibers exhibited the type IV isotherms according to IUPAC classification, confirming the preservation of mesoporosity. Moreover, the shapes of the hysteresis loops were of type H2 at relative pressure between 0.4 and 0.8, which was associated with the aggregation of primary particles giving rise to ink-bottle pores^29, 30^. Importantly, the SSA of the membranes increased at first and then decreased with increasing Zr concentration from 0 to 20 mol% (Table S4), and the TZ-10 displayed the largest SSA of 38.8 m^2^ g^−1^ due to the smallest particle size. In addition, the pore size distribution of nanofibers was analysed based on the Barrett-Joyner-Halenda (BJH) method. Figure 4b shows TiO_2_ nanofibers had mesopores with an average pore size of about 10 nm, which were mainly caused by the obvious surface defects in the fibers (Fig. 1a) and could be further confirmed by the corresponding HR-TEM image (Figure S7a); while the TZ-10 nanofibers possessed smaller average pore size of 4.8 nm and the surface was smooth without obvious defects (Figure S7b) ascribed to the dramatically decreased crystal size and the more dense-packing structure of grains. These results provide evidence that Zr is a kind of effective structural stabilizers, which could inhibit the crystal growth and decrease the surface defects of TiO_2_ nanofibers. Therefore, it should be anticipated that the reduced crystal size, enlarged SSA, and uniform mesoporous structure with small pore size contribute to improve the mechanical properties and photocatalytic performance of TZ membranes.

### Mechanical properties of TZ nanofibrous membranes

**Figure 5:**
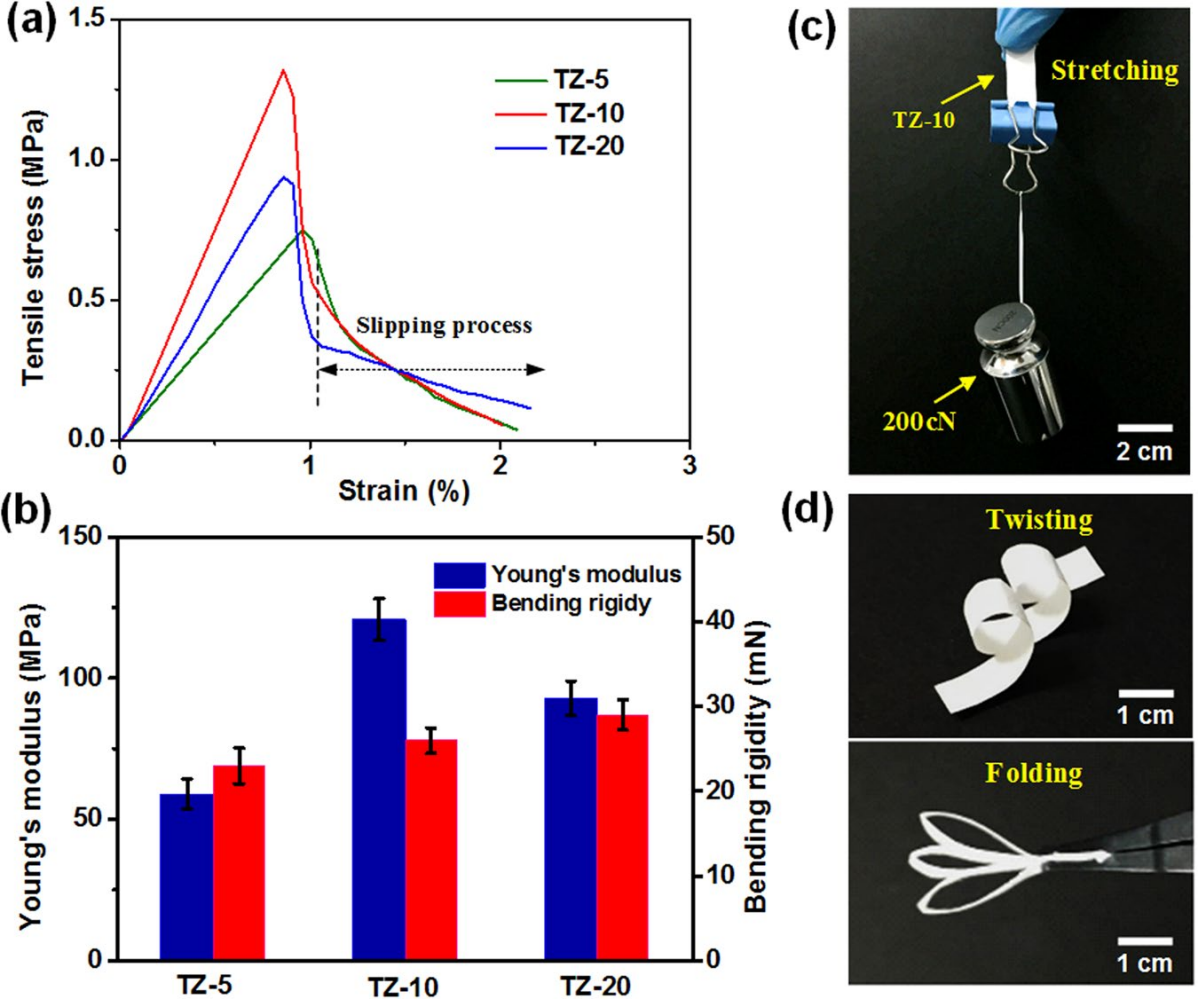
(**a**) Tensile stress-strain curves, and (**b**) Young’s modulus and bending rigidity of TZ-5, TZ-10, and TZ-20 fibrous membranes. (**c**,**d**) Photographs depicting the softness of TZ-10 fibrous membranes: it can be stretched, twisted and folded without damage.

The mechanical properties, including tensile strength, bending rigidity and durability, are indispensable for the practical application of photocatalytic nanofibrous membranes. Figure 5a presents the typical tensile stress-strain curves of TZ fibrous membranes containing various Zr contents. It demonstrated that the tensile strength increased from 0.75 to 1.32 MPa with the content of Zr increased from 5 to 10 mol% owing to the decreased grain size and pore size. However, further increasing Zr to 20 mol% resulted in the decrease of tensile strength to 0.94 MPa due to the coarsening of nanocrystals after doping with excessive Zr. As expected, the Young’s modulus of various samples were 59.0, 120.7, and 92.9 MPa, respectively, which showed the same change trend with the tensile strength (Fig. 5b). All samples exhibited a linear elastic behaviour in the early stages of the tensile loading and subsequently underwent a finite stress drop instead of catastrophic fracture when the stress achieved its maximum yield value. Further increasing the tensile loading, the membranes would continue to deform and gradually lead to an extended strain of 2% before failure, such a failure strain is approximately 10 times higher than that of traditional ceramics (0.1–0.2%) at room temperature^31^. This phenomenon could be ascribed to the slippage of nanofibers in the membranes, which has been demonstrated by SEM images during the entire stretching process (Figure S8). When a small external stress was loaded, randomly oriented fibers started to align and then some of them were broken, which led to little elongation. With continual increasing of tensile stress, these nanofibers with high aspect ratio slipped along the stress direction until they were completely separated, which would then inhibit the catastrophic fracture of inorganic fibrous membranes and resulted in a further increase in the elongation.

Generally, the membranes with lower bending rigidity exhibited better softness^32^, therefore the bending rigidity was measured to compare the softness of various membranes. As displayed in Fig. 5b, the bending rigidity of the TZ membranes increased slightly with the increment of Zr content due to the gradually increased fiber diameter^33^. Although the TZ-5 membranes showed the best softness, its durable bending ability was poor compared with the TZ-10 membranes, which have been confirmed via the repeated bending deformation test (Figure S9). After 200 cycles of bending to a radius of 2 mm, the breakage was found in the middle of the TZ-5 membranes, while the TZ-10 membranes still had the perfect appearance without broken due to its better mechanical strength. Significantly, the TZ-10 membranes (26 mN) are even softer than nonwovens (38 mN) and tissue paper (86 mN) (Figure S10). More interestingly, the TZ-10 membranes (width of 10 mm and thickness of 0.2 mm) could withstand a weight of 200 cN (Fig. 5c), and the membranes also could be severely twisted and folded without any mechanical rupture (Fig. 5d).

**Figure 6:**
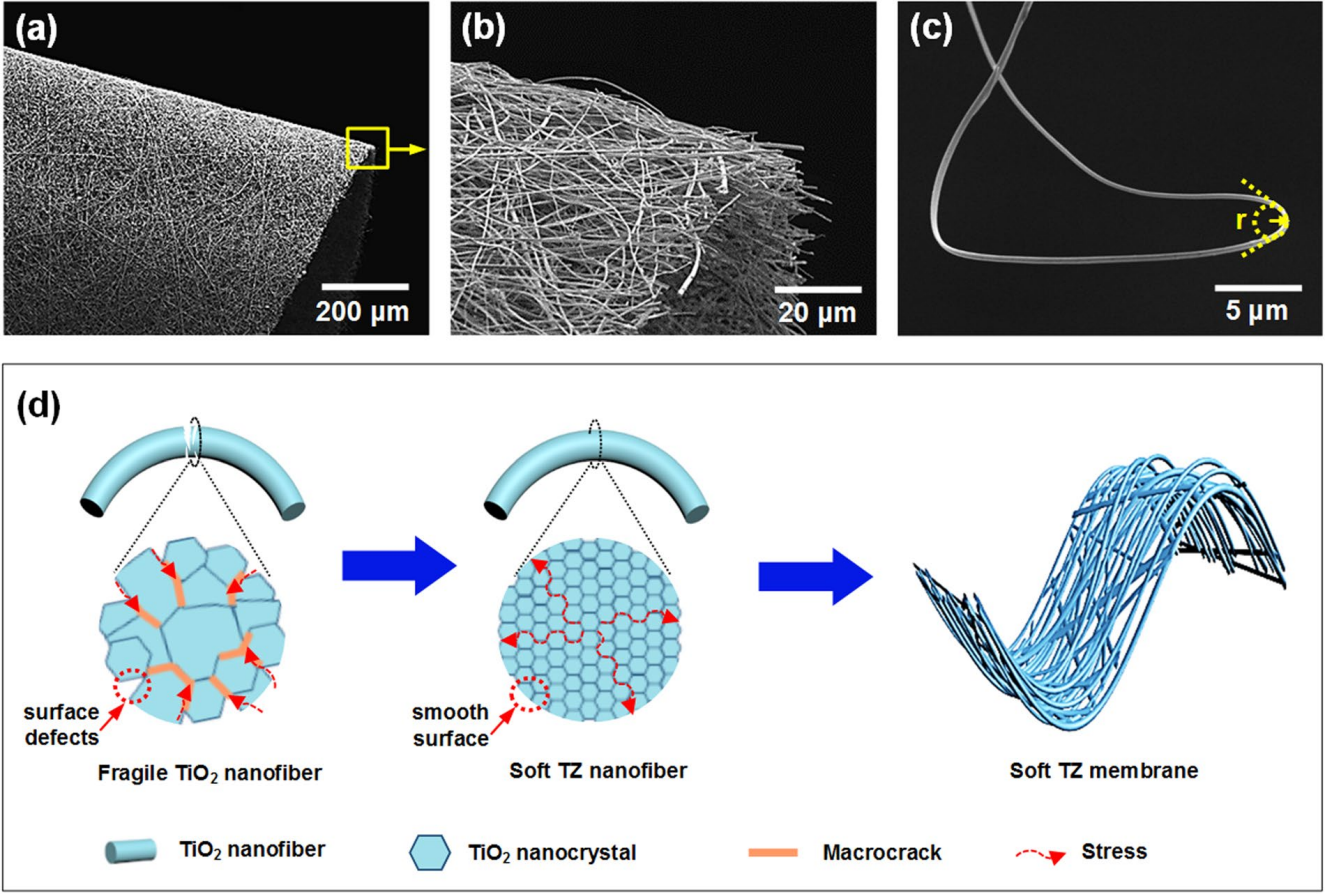
(**a**) SEM image of the bended TZ-10 nanofibrous membranes. (**b**) The high magnification SEM image of selected area in (**a**). (**c**) SEM image of bended single TZ-10 nanofiber. (**d**) Schematic illustration showing the probable mechanism of the softness of TZ membranes.

The superior mechanical properties of TZ-10 nanofibrous membranes can be further confirmed through directly observing the microstructural change after bending deformation. As illustrated in Fig. 6a and b, the membrane remained a structural integrity and continuous network without any fracture after bending down to a radius of 10 μm. Furthermore, we found that the single nanofiber (Fig. 6c) even could tolerate an extreme deformation with bending radius r < 1 μm, which endows the fibrous membranes with remarkable mechanical flexibility over traditional ceramic materials. Consequently, a plausible bending deformation mechanism for the TZ fibrous membranes was proposed (Fig. 6d) based on systematic analysis of the nanostructures of membranes. As the building blocks of fibrous membranes, the polycrystalline nanofibers with good bend ability are the precondition to construct the soft membranes. For pure TiO_2_ nanofiber, the excessive crystallite growth during calcination process would produce numerous surface defects in the fiber, when this fiber is subjected to external force or load, the stress would condense on these defect areas, resulting in a catastrophic failure as macrocracks propagate through the whole fiber. Otherwise, for the TZ nanofiber, the addition of Zr plays an active role in enhancing the structure stability of TiO_2_ fiber, which can inhibit the crystal growth and decrease the surface defects and pore size; the smaller crystal size and more dense-packed grains correspond to an increased grain boundary, which could efficiently scatter and dissipate the stress^34, 35^, as a result, the crack initiation and propagation in the fiber could be suppressed. In addition, according to the Hall-Petch effect, polycrystalline nanofiber with ultrafine grains could tolerate severe deformation owing to the enhanced strength and superplasticity caused by grain-boundary sliding^35, 36^. Therefore, the TZ fiber could exhibit high softness even under an extreme deformation. On the other hand, the TZ membranes remained a well-interconnected and continuous netlike structure, which enable the membranes to maintain its structural integrity upon bending by releasing the external stress through the interconnected nanofibers, supporting their superior structural flexibility^36^.

### Photocatalytic activity

**Figure 7:**
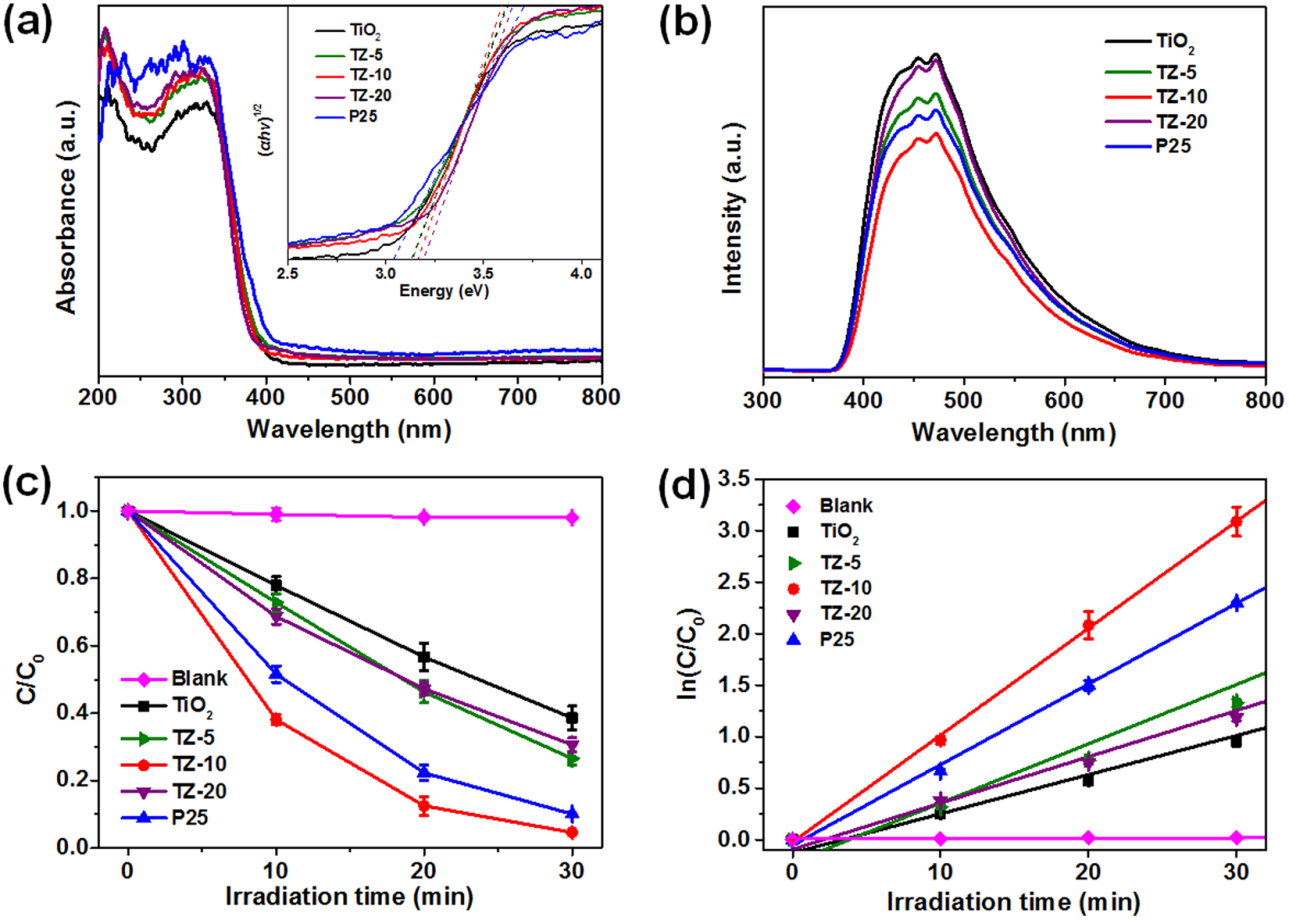
(**a**) UV-vis spectra and (**b**) PL spectra of TiO_2_, TZ-5, TZ-10, TZ-20, and P25. (**c**) Photocatalytic degradation performance and (**d**) kinetic linear fitting curves of MB over different samples under UV light irradiation. The inset showing the corresponding Kubelka-Munk transformed reflectance spectra to determine the indirect new bandgap values of the as-synthesized nanofibers.

The UV-vis diffuse reflectance spectra of photocatalysts are shown in Fig. 7a, indicating that all samples mainly absorb the UV light at wavelengths below 400 nm, which is in accordance with the intrinsic absorption of TiO_2_. Compared with the pure TiO_2_ nanofibers, the TZ nanofibers and P25 exhibit an increased adsorption in the UV region, this phenomenon would be reasonable considering the enhanced mesoporosity and SSA could promote light absorption and reflection; thus, would be enhanced the photocatalytic performance of the TiO_2_ nanofibers. Furthermore, the band gaps of the photocatalysts were calculated by the following formula^37^:

$$\alpha hv=A{(hv-{E}_{g})}^{2}$$

where *α* is the absorption coefficient, *A* is a constant, *hv* is the discrete photon energy, *E*_g_ is band energy. As exhibited in the inset of Fig. 7a, pristine TiO_2_ nanofibers exhibited a band gap about 3.13 eV, which was very close to the reported value of anatase TiO_2_^38^. The estimated band gaps of the TZ-5, TZ-10, and TZ-20 were 3.14, 3.17, and 3.19 eV, respectively. The slightly enlargement of band gap can be ascribed to the changes in their electronic structures after Zr^4+^ incorporation. In the case of P25, the band gap was calculated to be 3.04 eV, which was smaller than TiO_2_ and TZ nanofibers due to the mixed-phase effects of semiconductors^39^. Anyway, it is generally accepted that a larger band gap corresponds to more powerful redox ability, which may contribute to improve the photocatalytic performance^40–42^.

The photoluminescence (PL) spectra can be employed to investigate the electron-hole recombination after irradiation. As illustrated in Fig. 7b, all photocatalysts showed distinctive PL signals following excitation at a wavelength of 300 nm. The peaks at approximately 454 and 472 nm could be assigned to the emission of the charge transfer transition from oxygen vacancy trapped electrons^43, 44^. Interestingly, the TZ samples exhibited lower emission intensities than TiO_2_, indicating that Zr doping can effectively supress charge carrier recombination. This phenomenon can be explained by that the surface hydroxyl groups favor for the trapping of the holes to produce hydroxyl radicals, which can enhance the separation efficiency of photogenerated electron-hole pairs. The surface hydroxyl groups can be confirmed by the FT-IR spectra of undoped TiO_2_ and TZ-10 samples (Figure S11); both samples showed two bands at 3432 cm^−1^ and 1620 cm^−1^, corresponding to the stretching and bending vibrations of O-H bond^8, 45^. Obviously, the intensity of the bands of TZ-10 was much stronger than those of TiO_2_, implying TZ-10 possessed more surface hydroxyl groups. On the other hand, the substitution of Ti^4+^ by Zr^4+^ leads to lattice strain and thus to structural defects such as vacancies, the resultant defects can act as trapping centres to inhibit charge recombination^22, 46^. However, when the Zr concentration exceed the optimum value (in this paper is 10 mol%), the rate of charge recombination will increase because the distance between separating electron and hole is too small, resulting in a decreased number of effective charge carriers^45^. Hence, the TZ-10 membranes are anticipated to exhibit better performances in the photocatalytic applications.

The transient photocurrent responses and electrochemical impedance spectra (EIS) of the samples were performed to further investigate the separation and transfer of electron-hole pairs. Generally, higher photocurrent reflects higher separation efficiency for the photoinduced electron-hole pairs, and thus represents higher photoactivity^47^. As can be seen from Figure S12a, the TZ-10 photocatalyst exhibited the highest transient photocurrent among these photocatalysts, indicating that the suitable Zr doping in TiO_2_ nanofibers can effectively suppresses the recombination of photoinduced electrons and holes. Figure S12b shows the Nyquist plots of EIS for various photocatalysts. The semicircle in the Nyquist plot corresponds to the charge transfer resistance at the electrode-sample interface^48^. Compared with other photocatalysts, the TZ-10 photocatalyst shows smaller semicircle, which suggested that a faster interfacial charge transfer.

The photocatalytic activity of the as-fabricated TiO_2_ and TZ fibrous membranes was studied by measuring their photodegradation performance towards MB in aqueous solutions under UV light irradiation, and the data of commercial P25 nanoparticles was also given for comparison. It can be seen from Fig. 7c that self-decomposition of MB solution (<5%) could be neglected under UV light and the membranes could achieve adsorption-desorption equilibrium within 60 min (Figure S13), thus ensuring the accuracy of the experiment results. After photo-irradiation for 30 min, the degradation of MB dye was approximately 61.4, 73.5, 95.4, and 69.3% for TiO_2_, TZ-5, TZ-10, and TZ-20 fibrous membranes, respectively. Moreover, the activity of TZ-10 membranes was higher than that of P25 (90.0%). For a better comparison of the photocatalytic efficiency of the as-prepared photocatalysts, the kinetic analysis of degradation of MB was investigated by using the pseudo-first-order kinetics model as follows^49^:

$$ln({C}_{{0}}/{C}_{t})=kt$$

where *C*_*0*_ and *C*_*t*_ (mg L^−1^) are the concentrations of MB at time 0 and *t* (min), respectively, and *k* (min^−1^) is the pseudo-first-order rate constant. The constants *k* of various samples were 0.038, 0.058, 0.104, 0.045, and 0.078 min^−1^ with high correlation coefficients (*R* ^2^) of 0.995, 0.960, 0.998, 0.994, and 0.998, respectively, indicating the photocatalytic behaviour of all the samples obeyed the pseudo-first-order reaction dynamics model. Furthermore, these results clearly verified that the TZ-10 fibrous membranes possessed the highest photocatalytic activity.

In order to explore the major active species generated during the photocatalytic oxidation reaction for TZ-10, three typical chemicals including isopropanol (IPA), benzoquinone (BQ), and ethylenediaminetetraacetic acid disodium salt (EDTA-2Na) were employed as the scavengers of hydroxyl radical (•OH), superoxide radical (•O^2−^), and hole (h^+^), respectively^47, 48^. As shown in Figure S14. EDTA-2Na had little effect on MB degradation, suggesting that few holes were involved in decomposition process. The degradation efficiency of MB decreased from 95.4% to 57.3% in the presence of BQ, indicating that moderate amounts of •O^2−^ were generated. Furthermore, the photocatalytic activity of TZ-10 could be greatly prevented by the addition of IPA, demonstrating that •OH were the predominant active species in MB photodegradation. The results revealed that the generated •OH and •O^2−^ should be responsible for the enhanced photooxidation properties towards MB degradation.

As discussed above, the enhanced photocatalytic performance of TZ fibrous membranes can be attributed to the following factors. Firstly, the preservation of mesoporous structures and high SSA promote the absorption of UV light within the photocatalyst and offer more surface active sites for reactants, thereby contributing to improve the photodegradation activity. Secondly, the addition of Zr into TiO_2_ suppresses the crystal growth of the anatase during calcination. The smaller anatase crystal size provide a shorter migration distance for photoinduced electron-hole pairs, thus facilitating faster accessibility to the reaction sites and enhancing the photocatalytic activity. Thirdly, the Zr^4+^ incorporation could obviously inhibit the loss of surface hydroxyl groups on the fiber, thus the preserved surface hydroxyl groups could effectively capture the photoinduced holes, and then produced active hydroxyl radicals with strong oxidizing property. More importantly, electron-hole recombination could be effectively inhibited through the formation of surface hydroxyl groups and suitable oxygen vacancies, consequently, leading to the enhancement of photocatalytic activity of TZ membranes.

**Figure 8:**
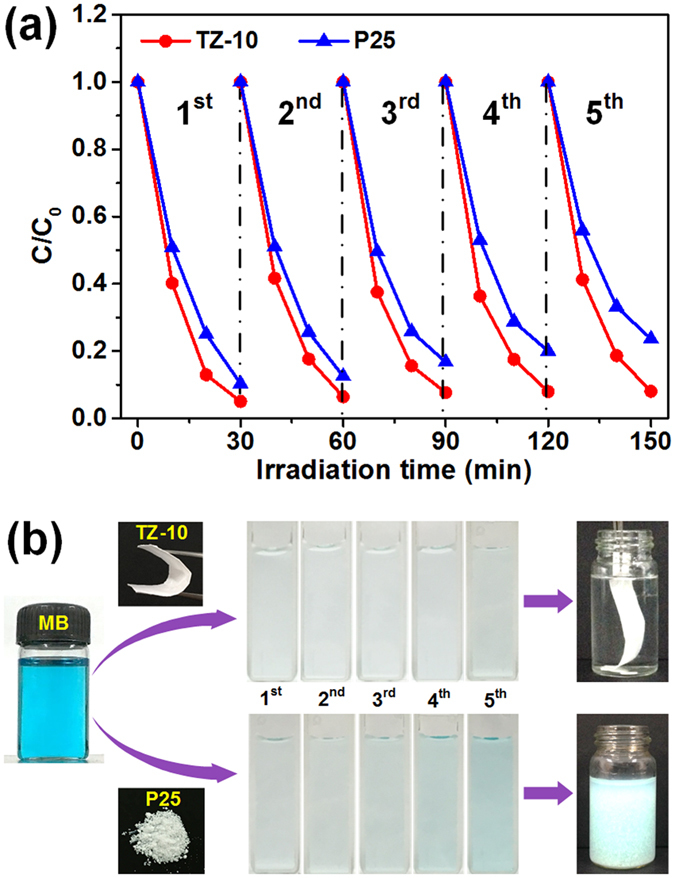
(**a**) Cycling photodegradation of MB over TZ-10 and TiO_2_ under UV light irradiation. (**b**) Photographs of MB solutions which undergo UV light photodegradation for 5 cycles with TZ-10 and P25.

The recyclability and stability of the photocatalysts are essential factors for the practical applications. To evaluate the degradation/regeneration capacity and investigate the structural stability during the entire process, we performed the reversibility of P25 nanoparticles and TZ-10 fibrous membranes for 5 times, respectively. Figure 8a shows the degradation degree of MB over P25 declines from 90% to 76.4% after 5 cycles, which demonstrated its poor stability. While the TZ-10 could still maintain high photocatalytic activity (92.1%) after 5 cycles, revealing the excellent reusability and photocatalytic activity retention capability of as-prepared fibrous membranes. More importantly, the membranes still exhibited good structural integrity after degradation/regeneration (Fig. 8b), and thus the membranes could be easily separated from the water for recycling using a tweezer without any tedious and time-consuming sedimentation process. In comparison, P25 nanoparticles tended to aggregate in the suspension during the photocatalytic cycles, which could only be separated by centrifugation. The XRD patterns of regenerated TZ-10 suggested that no obvious crystallinity loss or phase change was observed (Figure S15a). Additionally, the surface area of the TZ-10 decreased slightly from 38.8 m^2^ g^−1^ to 35.6 m^2^ g^−1^ and the mesoporous structure was preserved well after 5 cycles (Figure S15b). We attribute the excellent reversibility of TZ-10 photocatalyst to the stable anatase crystal structure and mesoporous structure, and the satisfactory mechanical properties, which could prevent the membranes from breaking up during the recycling process. Therefore, the above results suggested that the as-prepared soft TZ-10 fibrous membranes possessed excellent mechanical flexibility, efficient photocatalytic activity and good reusability, which could be applied as a high performance and easily recycled photocatalysts.

## Discussion

In summary, we have demonstrated a facile synthesis of soft TiO_2_ nanofibrous membranes by the combining of electrospinning and the Zr doping. The substitutional doping of Zr^4+^ could effectively inhibit the grain growth and reduce the surface defects and breaking point of nanofiber, which would endow the membranes with good tensile strength and bending resistance of 200 times. Moreover, the introduction of Zr^4+^ also would increase the SSA and suppress the electron-hole recombination, thereby improved the photocatalytic activity of as-prepared membranes towards MB. Under the optimized conditions, the resultant TZ-10 membranes exhibited good photocatalytic performance including an exceptional degradation degree of 95.4% within 30 min, fast degradation rate of 0.104 min^−1^ and good reusability in 5 cycles. More importantly, such membranes possessed better photodegradation activity than P25 nanoparticles and could be easily taken out from solution without using any cumbersome post-treatments. We anticipate that this work would provide a new strategy for the design and integration of multifunctional inorganic nanofibrous membranes with high mechanical flexibility and photocatalytic activity, which hold great potential for environmental remediation.

## Methods

### Materials

Titanium (IV) isopropoxide (TIP), zirconium acetate (Zr(Ac)_4_), polyvinylpyrrolidone (PVP) (*M*_*w*_ = 1,300,000), acetic acid (99.5%), and absolute ethanol (99.5%) were all purchased from Aladdin Chemical Co., Ltd., China. Deionized water with a resistivity of 18.2 MΩ was produced by a water purification system. All the initial chemicals were used without further purification.

### Preparation of soft TZ nanofibrous membranes

The TZ fibrous membranes were fabricated by combining the electrospinning technique and the subsequent calcination process described as follow: Firstly, 0.5 g PVP was dissolved in a mixed solution of 8.0 g absolute ethanol and 4.0 g acetic acid with vigorous stirring for 2 h. Then, an appropriate amount of TIP and Zr(Ac)_4_ were slowly added into the solution and stirring in an ice-water bath for 30 min to produce a transparent and homogeneous solution. The molar ratio of Zr relative to the total abundance of Ti and Zr ranged from 5, 10 to 20 mol%, which were labeled as TZ-5, TZ-10, and TZ-20, respectively. The electrospinning process was performed by using a DXES-3 spinning apparatus (SOF Nanotechnology Co., Ltd., China) with a stable flow rate of 1.5 mL h^−1^ and an applied voltage of 15 kV. All the precursor fibrous membranes were collected on a nonwoven fabric covering the roller collector with rotating speed of 50 rpm. During the entire electrospinning process, the ambient temperature and relative humidity were maintained at 25 ± 2 °C and 50 ± 2%, respectively. Finally, the as-spun hybrid fibrous membranes were annealed at 600 °C in air for 1 h with a heating rate of 2 °C min^−1^. The TGA curves in Figure S1 suggested that the organic components of hybrid fibers have been removed completely by heating to 600 °C. For comparative studies, pure TiO_2_ nanofibers were prepared by the same procedure as mentioned above.

### Characterization

Thermo gravimetric analysis (TGA) of hybrid fibrous membranes was performed on a thermogravimetric analyzer (SDT Q600) from 25 to 800 °C with a temperature increase rate of 10 °C min^−1^ under air atmosphere. The morphologies and microstructure of fibrous membranes were examined by a field emission scanning electron microscopy (FE-SEM, S-4800) and a high-resolution transmission electron microscopy (HRTEM, JEM-2100F). The crystal phase properties of samples were analyzed by X-ray diffraction (XRD, D/Max-2550 PC) with Cu Kα radiation (λ = 1.5406 Å). X-ray photoelectron spectroscopy (XPS) analysis was carried out on a PHI 5000 C ESCA system with Mg Kα source. The Brunauer-Emmet-Teller (BET) surface area and porous structures were evaluated using N_2_ adsorption at 77 K on a surface area analyzer (ASAP 2020). In order to evaluate the mechanical properties of the resultant TZ membranes, the tensile strength of the membranes (size of 5 × 0.3 cm^2^ and thickness of 50 ± 5 μm) was measured on a tensile tester (XQ-1C) according to the standard (ISO 1798:2008), and the bending rigidity of the membranes (size of 10 × 10 cm^2^ and thickness of 50 ± 5 μm) was conducted on a softness tester (RRY-1000) according to the standard (ASTM D 2923-95), the optical images of mechanical test instruments were presented in Figure S2. The absorption spectra of photocatalysts was recorded using a UV-vis spectrometer (U-3900) with an integrating sphere. Fourier transform infrared (FT-IR) spectroscopy was performed on a Nicolet iS10 spectrometer in the range of 4000–500 cm^−1^. Photoluminescene (PL) spectra of photocatalysts was recorded by a fluorescence spectrophotometer (F-4600) using an excitation wavelength of 300 nm.

### Photocatalytic activity evaluation

The photocatalytic degradation of MB was employed to characterize the reactivities of TZ nanofibers, pure TiO_2_ nanofibers, and P25 nanoparticles. In a typical process, 20 mg photocatalysts were first added into 100 mL MB solution (10 mg L^−1^), and then the solution was slightly stirred for 30 min in the dark to reach the adsorption-desorption equilibrium between the reactants and catalysts. The photocatalytic degradation was triggered under the irradiation from a 24 W ultraviolet lamp with wavelength λ = 254 nm. Afterwards, at given intervals of illumination, the concentrations of remaining MB was monitored using a UV-vis spectrophotometer (PG2000-Pro) at λ = 664 nm. The active species generated during the photocatalytic oxidation reaction were detected by adding 1 mM isopropanol (IPA), 1 mM ethylenediaminetetraacetic acid disodium salt (EDTA-2Na), and 1 mM benzoquinone (BQ) as scavengers. In order to measure the reversibility of the soft TZ fibrous membranes, the reacted membranes were rinsed with pure water, and dried in an oven at 100 °C for 1 h to obtain the regenerated photocatalysts. Then, the membranes were reused for another cycle by using the same procedure mentioned above.

### Photoelectrochemical measurements

Photocurrent and electrochemical impedance spectrum (EIS) were measured on CHI-660E electrochemical workstation by using a standard three-electrode system. Platinum wire and Ag/AgCl electrode were employed as the counter electrode and reference electrode, respectively. To prepare the working electrode, 5 mg photocatalyst was mixed with 5 mL ethanol to form slurry, which was then coated onto fluorine-doped tin oxide (FTO) glass electrode with an effective area of 1 × 1 cm^2^_._ Then the as-prepared working electrode was immersed in 0.5 M Na_2_SO_4_ electrolyte solution and irradiated under a 24 W UV-light (λ = 254 nm).

## Electronic supplementary material

Soft Zr-doped TiO2 Nanofibrous Membranes with Enhanced Photocatalytic Activity for Water Purification

## References

[1] Matlock MM, Howerton BS, Atwood DA (2002). Chemical precipitation of heavy metals from acid mine drainage. Water. Res.

[2] Wen Q (2013). Flexible inorganic nanofibrous membranes with hierarchical porosity for efficient water purification. Chem. Sci.

[3] Lee M, Merle T, Rentsch D, Canonica S, von Gunten U (2017). Abatement of polychoro-1,3-butadienes in aqueous solution by ozone, UV photolysis, and advanced oxidation processes (O_3_/H_2_O_2_ and UV/H_2_O_2_). Environ. Sci. Technol..

[4] Schneider J (2014). Understanding TiO_2_ photocatalysis: mechanisms and materials. Chem. Rev..

[5] Kumar SG, Rao KSRK (2017). Comparison of modification strategies towards enhanced charge carrier separation and photocatalytic degradation activity of metal oxide semiconductors (TiO_2_, WO_3_ and ZnO). Appl. Surf. Sci..

[6] Juma A (2016). Zirconium doped TiO_2_ thin films deposited by chemical spray pyrolysis. Appl. Surf. Sci..

[7] Strini A (2015). *In-situ* anatase phase stabilization of titania photocatalyst by sintering in presence of Zr^4+^ organic salts. Appl. Surf. Sci..

[8] Chen X, Wang X, Fu X (2009). Hierarchical macro/mesoporous TiO_2_/SiO_2_ and TiO_2_/ZrO_2_ nanocomposites for environmental photocatalysis. Energy Environ. Sci..

[9] Fu C (2016). Photocatalytic enhancement of TiO_2_ by B and Zr co-doping and modulation of microstructure. Appl. Surf. Sci..

[10] Im JS, Kim MI, Lee Y-S (2008). Preparation of PAN-based electrospun nanofiber webs containing TiO_2_ for photocatalytic degradation. Mater. Lett..

[11] Su C, Tong Y, Zhang M, Zhang Y, Shao C (2013). TiO_2_ nanoparticles immobilized on polyacrylonitrile nanofibers mats: a flexible and recyclable photocatalyst for phenol degradation. RSC Adv.

[12] Leyland NS (2016). Highly efficient F, Cu doped TiO_2_ anti-bacterial visible light active photocatalytic coatings to combat hospital-acquired infections. Sci. Rep.

[13] Xue H (2016). Floating photocatalyst of B-N-TiO_2_/expanded perlite: a sol-gel synthesis with optimized mesoporous and high photocatalytic activity. Sci. Rep.

[14] Ma Z (2013). Luffa-sponge-like glass-TiO_2_ composite fibers as efficient photocatalysts for environmental remediation. ACS Appl. Mater. Interfaces.

[15] Si Y, Yu J, Tang X, Ge J, Ding B (2014). Ultralight nanofibre-assembled cellular aerogels with superelasticity and multifunctionality. Nat. Commun..

[16] Si Y (2015). Superelastic and superhydrophobic nanofiber-assembled cellular aerogels for effective separation of oil/water emulsions. ACS Nano.

[17] Zhang L (2017). Electrospun titania nanofibers segregated by graphene oxide for improved visible light photocatalysis. Appl. Catal. B.

[18] Wang X, Xi M, Fong H, Zhu Z (2014). Flexible, transferable, and thermal-durable dye-sensitized solar cell photoanode consisting of TiO_2_ nanoparticles and electrospun TiO_2_/SiO_2_ nanofibers. ACS Appl. Mater. Interfaces.

[19] Zhang R (2015). *In situ* synthesis of flexible hierarchical TiO_2_ nanofibrous membranes with enhanced photocatalytic activity. J. Mater. Chem. A.

[20] Li W, Wang Y, Ji B, Jiao X, Chen D (2015). Flexible Pd/CeO_2_-TiO_2_ nanofibrous membrane with high efficiency ultrafine particulate filtration and improved CO catalytic oxidation performance. RSC Adv.

[21] Liu Z (2014). Flexible polyaniline-coated TiO_2_/SiO_2_ nanofiber membranes with enhanced visible-light photocatalytic degradation performance. J. Colloid Interface Sci..

[22] Schiller R, Weiss CK, Landfester K (2010). Phase stability and photocatalytic activity of Zr-doped anatase synthesized in miniemulsion. Nanotechnology.

[23] Wang J (2013). Doping behavior of Zr^4+^ ions in Zr^4+^-doped TiO_2_ nanoparticles. J. Phys. Chem. C.

[24] Chang S, Doong R (2006). Characterization of Zr-doped TiO_2_ nanocrystals prepared by a nonhydrolytic sol-gel method at high temperatures. J. Phys. Chem. B.

[25] Gao B, Lim TM, Subagio DP, Lim T-T (2010). Zr-doped TiO_2_ for enhanced photocatalytic degradation of bisphenol A. Appl. Catal. A.

[26] Zhang P (2014). Structure of nitrogen and zirconium co-doped titania with enhanced visible-light photocatalytic activity. ACS Appl. Mater. Interfaces.

[27] Huang Q (2013). Photocatalytic decomposition of gaseous HCHO by Zr_x_Ti_1-x_O_2_ catalysts under UV-vis light irradiation with an energy-saving lamp. J. Mol. Catal. A-Chem..

[28] Cao YQ (2009). Structure and phase transition behavior of Sn^4+^-doped TiO_2_ nanoparticles. J. Phys. Chem. C.

[29] Xu FY, Xiao W, Cheng B, Yu JG (2014). Direct Z-scheme anatase/rutile bi-phase nanocomposite TiO_2_ nanofiber photocatalyst with enhanced photocatalytic H_2_-production activity. Int. J. Hydrogen Energ..

[30] Fu JW, Cao SW, Yu JG, Low JX, Lei YP (2014). Enhanced photocatalytic CO_2_-reduction activity of electrospun mesoporous TiO_2_ nanofibers by solvothermal treatment. Dalton Trans..

[31] Han XD (2007). Low-temperature *in situ* large strain plasticity of ceramic SiC nanowires and its atomic-scale mechanism. Nano Lett..

[32] Mao X (2016). Brittle-flexible-brittle transition in nanocrystalline zirconia nanofibrous membranes. CrystEngComm.

[33] Mao X, Bai Y, Yu J, Ding B, Ferreira J (2016). Flexible and highly temperature resistant polynanocrystalline zirconia nanofibrous membranes designed for air filtration. J. Am. Ceram. Soc..

[34] Wu ZX, Zhang YW, Jhon MH, Srolovitz DJ (2013). Anatomy of nanomaterial deformation: Grain boundary sliding, plasticity and cavitation in nanocrystalline Ni. Acta Mater..

[35] Meyers MA, Mishra A, Benson DJ (2006). Mechanical properties of nanocrystalline materials. Prog. Mater. Sci..

[36] Huang S (2014). A flexible and transparent ceramic nanobelt network for soft electronics. NPG Asia Mater.

[37] Zhang ZY (2013). Hierarchical assembly of ultrathin hexagonal SnS_2_ nanosheets onto electrospun TiO_2_ nanofibers: enhanced photocatalytic activity based on photoinduced interfacial charge transfer. Nanoscale.

[38] Pei CC, Leung WWF (2013). Enhanced photocatalytic activity of electrospun TiO_2_/ZnO nanofibers with optimal anatase/rutile ratio. Catal. Commun..

[39] Scanlon DO (2013). Band alignment of rutile and anatase TiO_2_. Nat. Mater..

[40] Chen L, Yang S, Mader E, Ma PC (2014). Controlled synthesis of hierarchical TiO_2_ nanoparticles on glass fibres and their photocatalytic performance. Dalton Trans..

[41] Yu JC (2002). Effects of F^-^ doping on the photocatalytic activity and microstructures of nanocrystalline TiO_2_ powders. Chem. Mater..

[42] Kumar SG, Devi LG (2011). Review on modified TiO_2_ photocatalysis under UV/visible light: selected results and related mechanisms on interfacial charge carrier transfer dynamics. J. Phys. Chem. A.

[43] Lin H, Wang X (2016). Epitaxy of radial high-energy-facetted ultrathin TiO_2_ nanosheets onto nanowires for enhanced photoreactivities. Adv. Funct. Mater..

[44] Xie Y, Zhang X, Ma P, Wu Z, Piao L (2015). Hierarchical TiO_2_ photocatalysts with a one-dimensional heterojunction for improved photocatalytic activities. Nano Res.

[45] Wang YM (2004). Preparation and photocatalytic properties of Zr^4+^-doped TiO_2_ nanocrystals. J. Mol. Catal. A-Chem..

[46] Goswami P, Ganguli JN (2013). Tuning the band gap of mesoporous Zr-doped TiO_2_ for effective degradation of pesticide quinalphos. Dalton Trans..

[47] Li X (2016). Hierarchical photocatalysts. Chem. Soc. Rev..

[48] Chen F (2017). Hierarchical assembly of graphene-bridged Ag_3_PO_4_/Ag/BiVO_4_ (040) Z-scheme photocatalyst: An efficient, sustainable and heterogeneous catalyst with enhanced visible-light photoactivity towards tetracycline degradation under visible light irradiation. Appl. Catal. B.

[49] Zhu LL (2015). Design of metal oxide-organic framework (MoOF) foam microreactor: solar-induced direct pollutant degradation and hydrogen generation. Adv. Mater..

