# Supplementary material for: Soft Zr-doped TiO2 Nanofibrous Membranes with Enhanced Photocatalytic Activity for Water Purification
Source: Sci Rep. 2017 May 9;7:1636. doi: 10.1038/s41598-017-01969-w (PMC5431652; doi:10.1038/s41598-017-01969-w)
Supplement: Supplementary file 1 — Soft Zr-doped TiO2 Nanofibrous Membranes with Enhanced Photocatalytic Activity for Water Purification [file 41598_2017_1969_MOESM1_ESM.pdf]

Supplementary information for

Soft Zr-doped TiO_2 Nanofibrous Membranes with Enhanced Photocatalytic Activity for Water Purification

Jun Song[1], Xueqin Wang[1], Jianhua Yan[2,3], Jianyong Yu[2,3], Gang Sun[2] & Bin Ding*[1,2,3]

[1]State Key Laboratory for Modification of Chemical Fibers and Polymer Materials, College of Materials Science and Engineering, Donghua University, Shanghai 201620, China

[2]Key Laboratory of Textile Science & Technology, Ministry of Education, College of Textiles, Donghua University, Shanghai 201620, China

[3]Nanofibers Research Center, Modern Textile Institute, Donghua University, Shanghai 200051, China

*Corresponding author: Prof. Bin Ding, E-mail address: binding@dhu.edu.cn

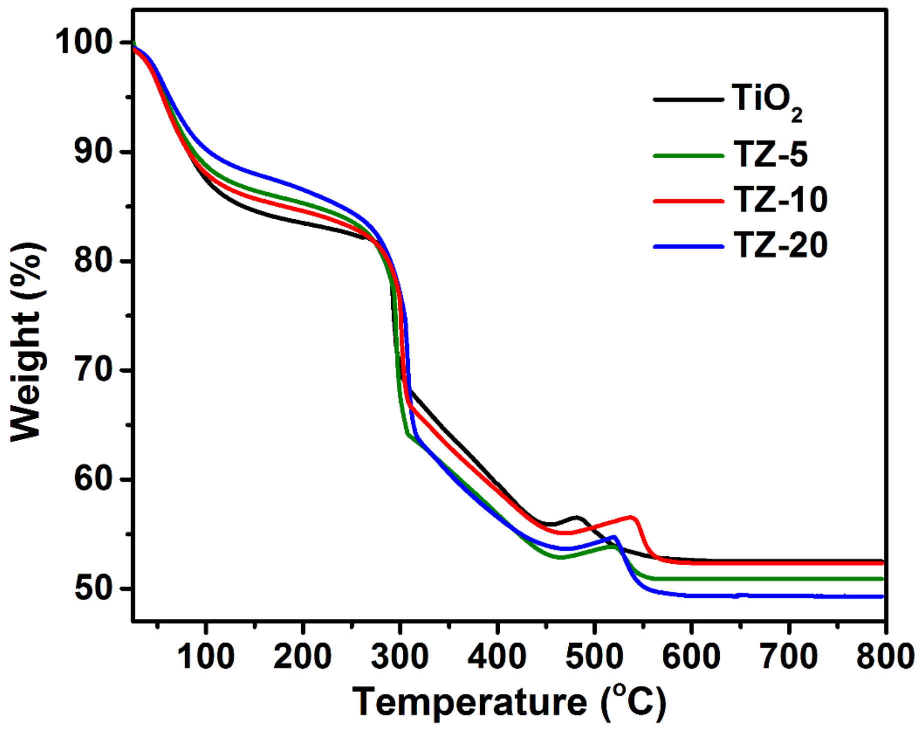

Figure S1. TGA cures of TiO_2, TZ-5, TZ-10, and TZ-20 fibrous mebranes.

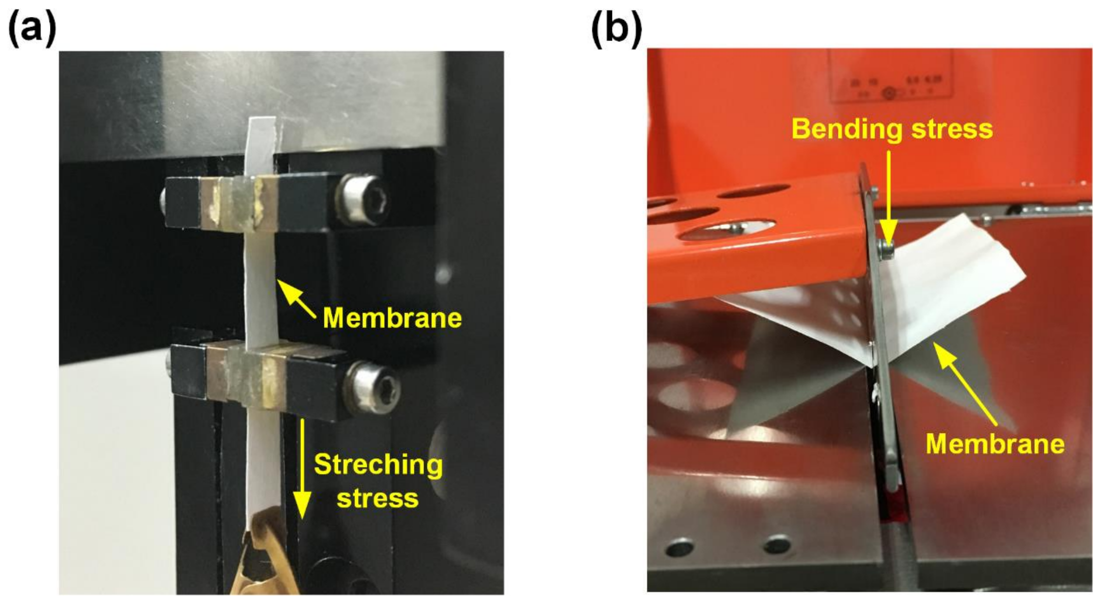

Figure S2. (a and b) The equipment for testing mechanical properties and bending rigidities, respectively.

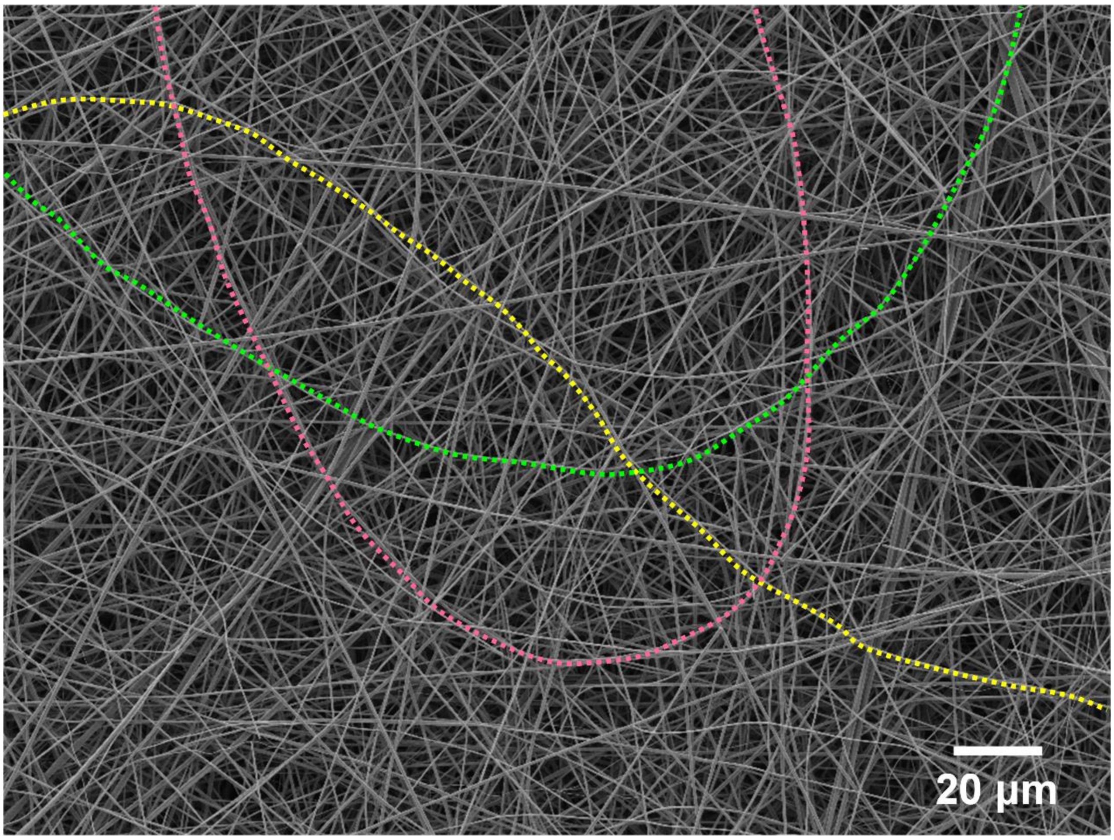

Figure S3. SEM image of electrospun TZ-10 membranes. The three highlighted lines clearly show that the nanofibers are continuous over 400 μm without any breakpoint.

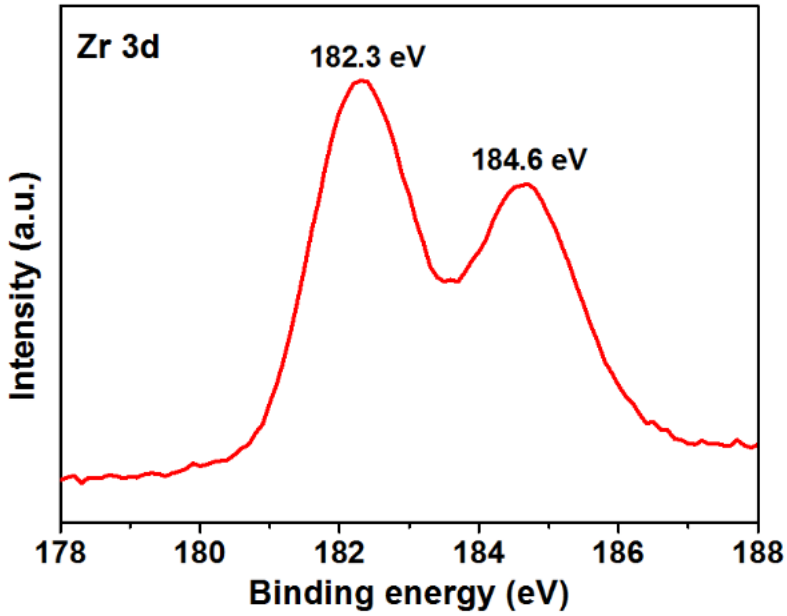

Figure S4. XPS spectrum of Zr 3d for TZ-10 membranes.

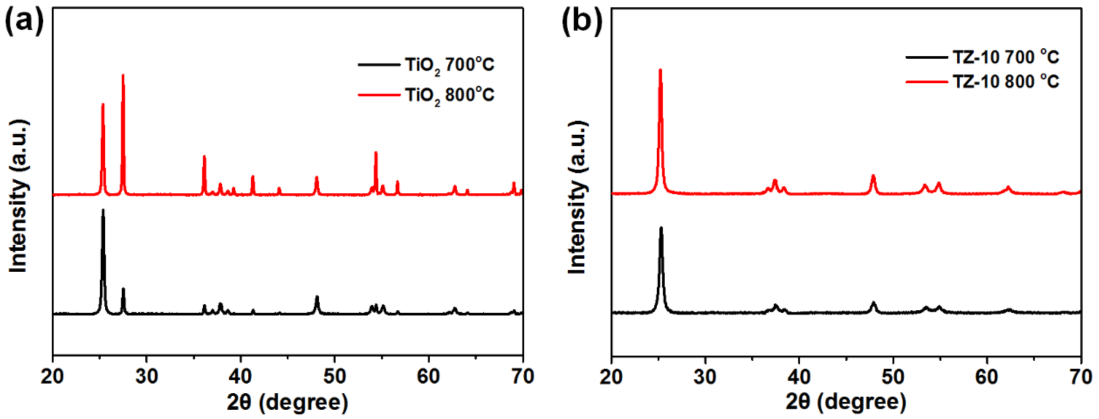

Figure S5. XRD patterns of (a) TiO_2 and (b) TZ-10 fibrous membranes calcinated at 700 and 800 °C.

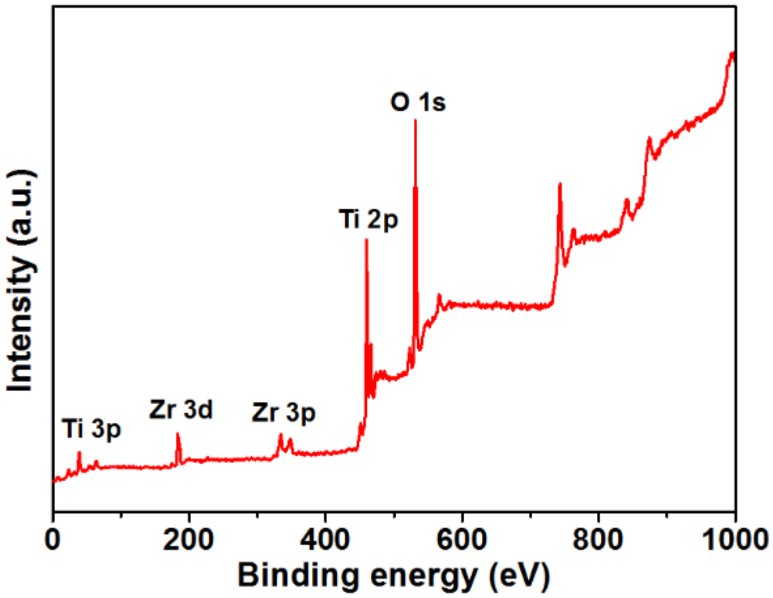

Figure S6. XPS spectrum of TZ-10 membranes.

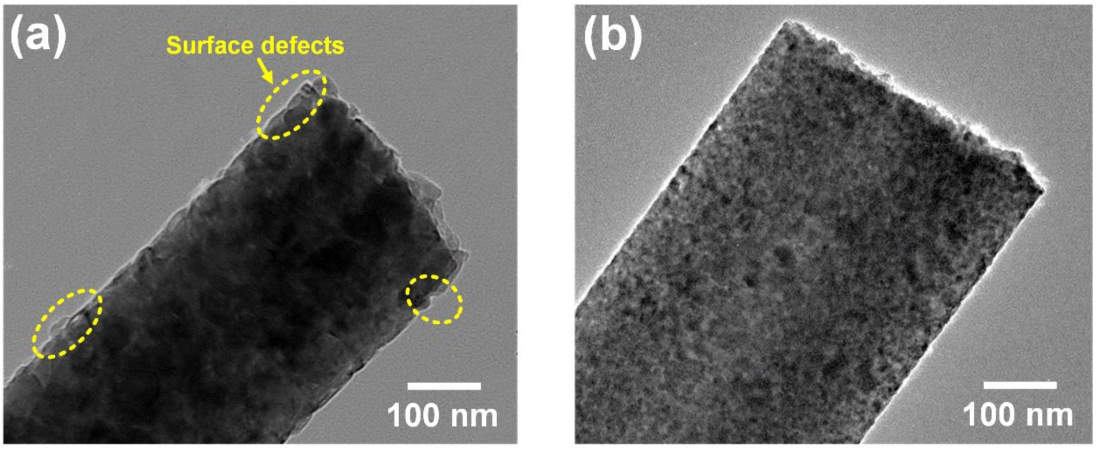

Figure S7. (a and b) HR-TEM images of TiO_2 and TZ-10 nanofiber, respectively.

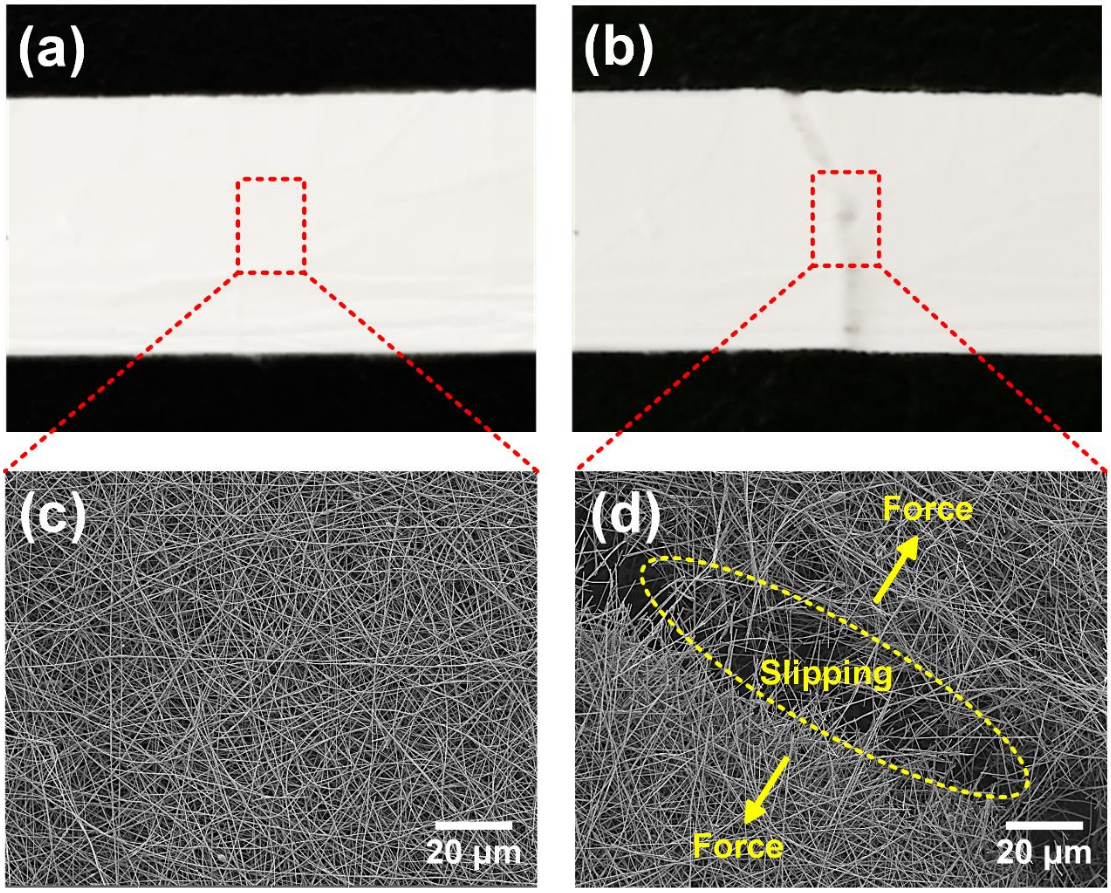

Figure S8. (a and b) Photographs of the TZ-10 membranes before tension and after failure, respectively. (c and d) SEM images of the TZ-10 membranes before tension and after failure, respectively.

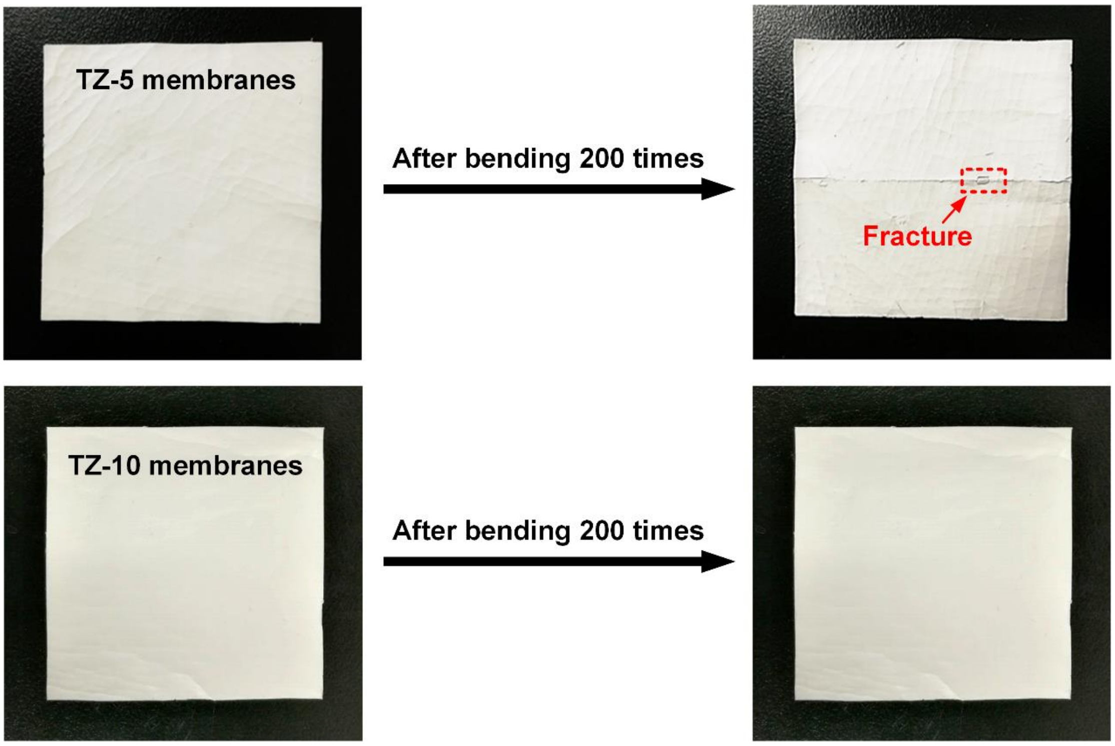

Figure S9. The photographs of TZ-5 and TZ-10 membranes before and after bending deformation of 200 times.

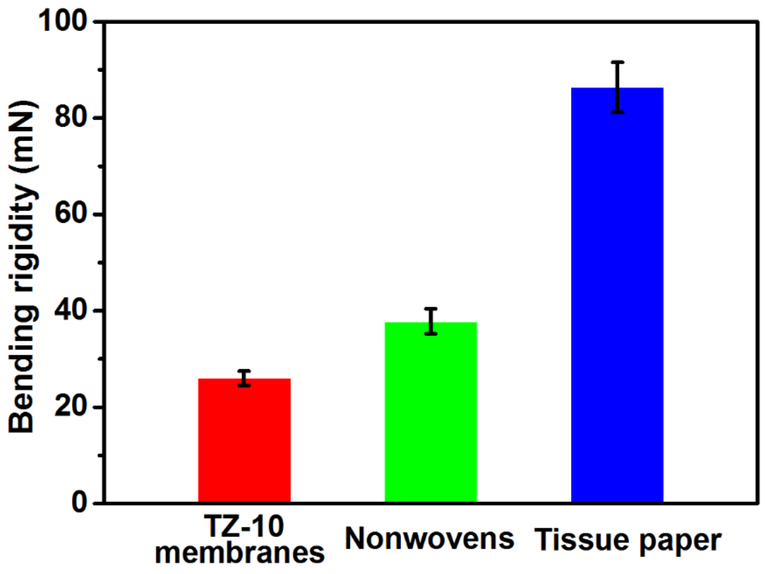

Figure S10. The bending rigidity of TZ-10 membranes, nonwovens, and tissue paper.

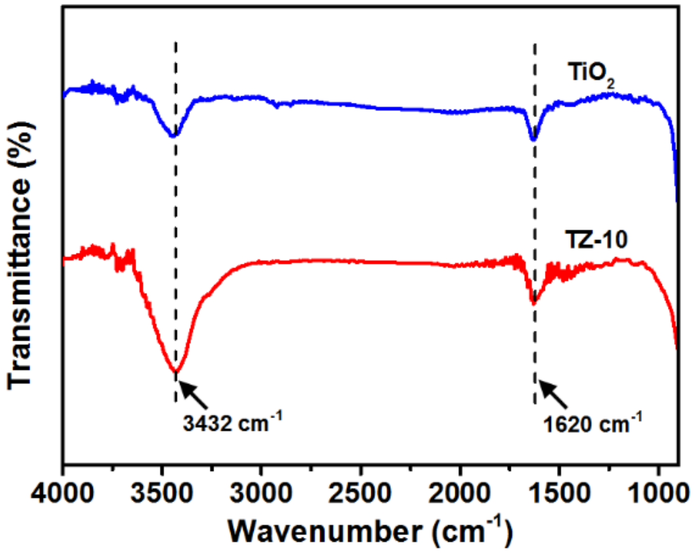

Figure S11. FTIR spectra of TiO_2 and TZ-10 membranes.

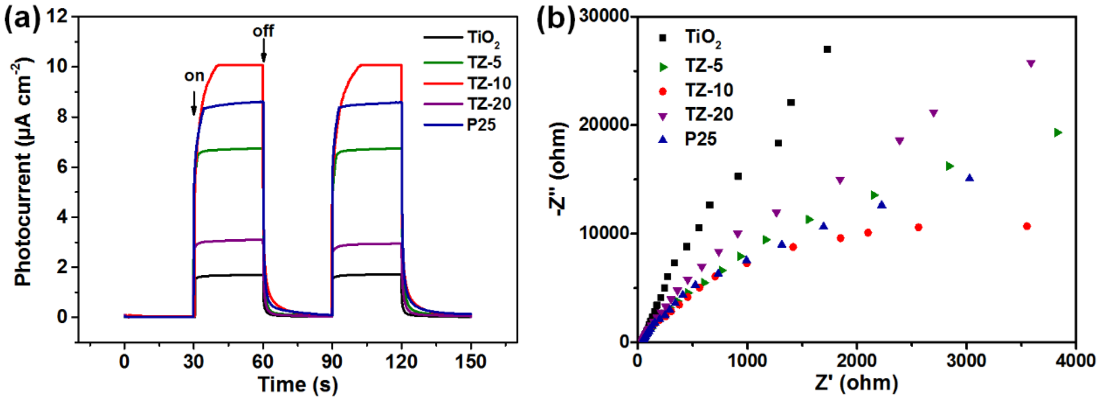

Figure S12. (a) Transient photocurrent responses and (b) Electrochemical impedance spectra of various samples.

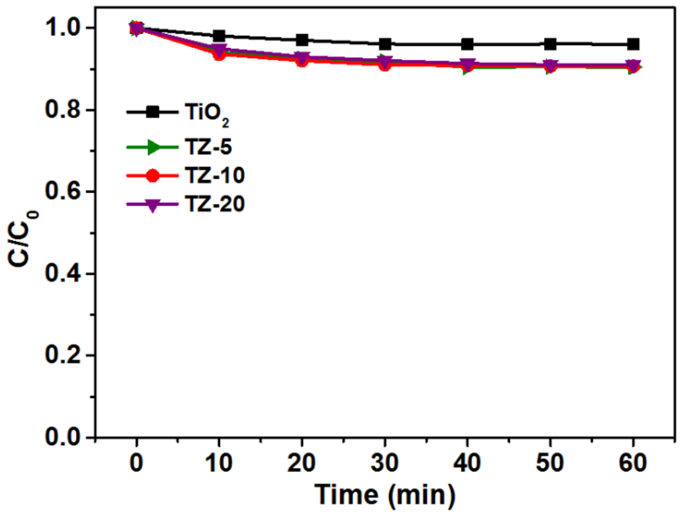

Figure S13. Adsorption performance of various membranes towards MB in the dark.

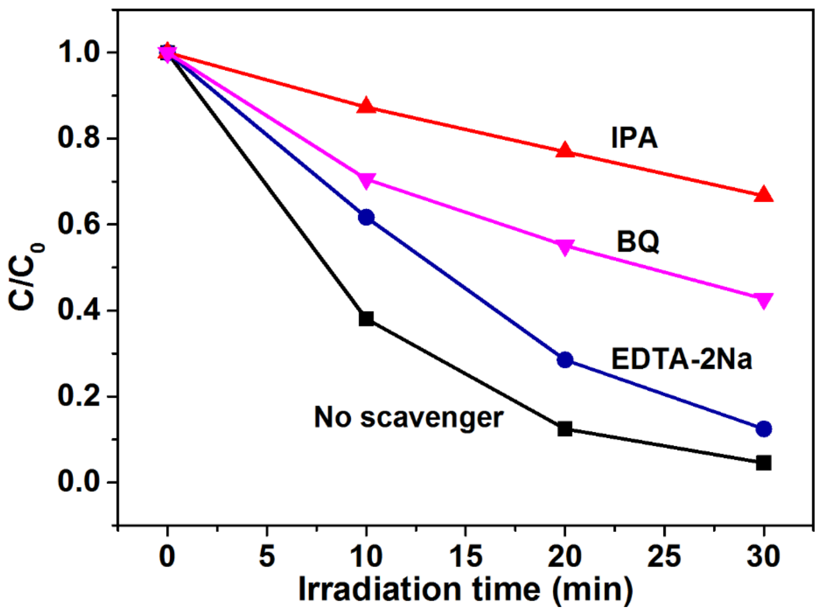

Figure S14. Trapping experiment of active species during the photocatalytic degradation of MB over TZ-10 fibrous membranes under UV light irradiation.

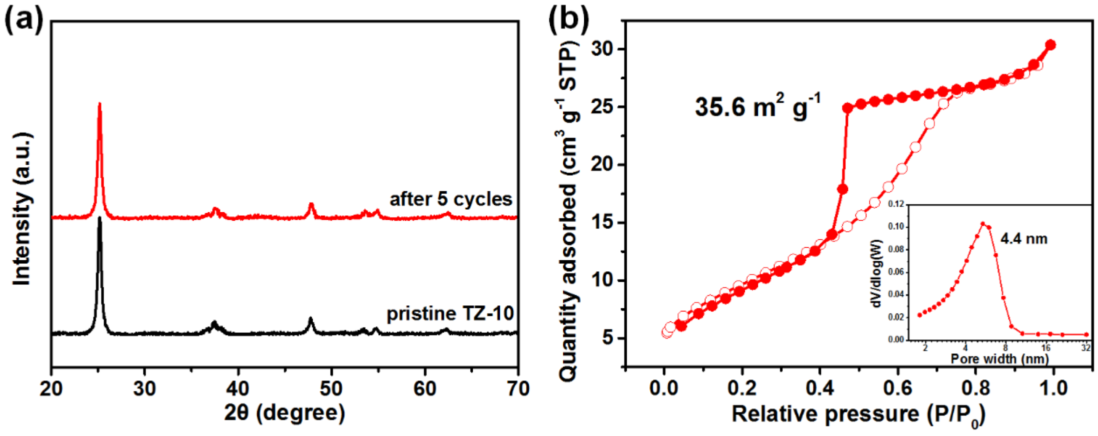

Figure S15. (a) N_2 adsorption-desorption isotherms and pore distribution of the recycled TZ-10 fibrous membranes. (b) XRD patterns of TZ-10 membranes before and after 5 cycles.

Table S1. Solutions properties of TiO_2 and TZ precursor solution

Samples	Viscosity (cps)	Conductivity (μS m^{-1})	Surface tension (mN m^{-1})
TiO_2	32	125	15
TZ-5	40	42	16
TZ-10	45	27	17
TZ-20	49	26	19

Table S2. Crystal phase, crystal size, lattice parameter, and cell volume for TiO_2 and TZ fibrous membranes.

Samples	Crystal phase	Crystal size (nm)	Lattice parameter (Å)	Cell volume (Å^3)
TiO_2	Anatase	29.1	a = b = 3.788, c = 9.519	136.59
TZ-5	Anatase	21.8	a = b = 3.793, c = 9.554	137.45
TZ-10	Anatase	17.5	a = b = 3.804, c = 9.591	138.78
TZ-20	Anatase	22.4	a = b = 3.814, c = 9.705	141.17

Table S3. Crystal phase and crystal size for TiO_2 and TZ-10 fibrous membranes annealed at 700 and 800 °C.

Samples	Annealing	Anatase (%)	Rutile (%)	Crystal size (nm)	
				Anatase	Rutile
TiO_2	700 °C	83.7	16.3	34.1	49.3
TiO_2	800 °C	46.9	53.1	44.9	60.0
TZ-10	700 °C	100	0	20.3	—
TZ-10	800 °C	100	0	26.6	—

Table S4. The summary of pore structure parameters of relevant membranes.

Samples	BET surface area (m^2 g^{-1})	BJH pore volume (cm^3 g^{-1})	Average pore size (nm)
TiO_2	7.0	0.011	10.0
TZ-5	37.1	0.053	6.2
TZ-10	38.8	0.043	4.8
TZ-20	30.3	0.031	4.6
